# The Potential Cost and Benefits of Raltegravir in Simplified Second-Line Therapy among HIV Infected Patients in Nigeria and South Africa

**DOI:** 10.1371/journal.pone.0054435

**Published:** 2013-02-15

**Authors:** Karen Schneider, Chidi Nwizu, Richard Kaplan, Jonathan Anderson, David P. Wilson, Sean Emery, David A. Cooper, Mark A. Boyd

**Affiliations:** 1 The Kirby Institute for Infection and Immunity in Society, University of New South Wales, Sydney, New South Wales, Australia; 2 Department of Medicine, Institute of Human Virology, University of Maryland School of Medicine, Baltimore, Maryland, United States of America; 3 Desmond Tutu HIV Foundation, Cape Town, South Africa; 4 St. Vincent's Hospital Centre for Applied Medical Research (SVH AMR), Sydney, New South Wales, Australia; 5 St. Vincent's Hospital, Sydney, New South Wales, Australia; University of Pittsburgh, United States of America

## Abstract

**Background:**

There is an urgent need to improve the evidence base for provision of second-line antiretroviral therapy (ART) following first-line virological failure. This is particularly the case in Sub-Saharan Africa where 70% of all people living with HIV/AIDS (PHA) reside. The aim of this study was to simulate the potential risks and benefits of treatment simplification in second-line therapy compared to the current standard of care (SOC) in a lower-middle income and an upper-middle income country in Sub-Saharan Africa.

**Methods:**

We developed a microsimulation model to compare outcomes associated with reducing treatment discontinuations between current SOC for second-line therapy in South Africa and Nigeria and an alternative regimen: ritonavir-boosted lopinavir (LPV/r) combined with raltegravir (RAL). We used published studies and collaborating sites to estimate efficacy, adverse effect and cost. Model outcomes were reported as incremental cost effectiveness ratios (ICERs) in 2011 USD per quality adjusted life year ($/QALY) gained.

**Results:**

Reducing treatment discontinuations with LPV/r+RAL resulted in an additional 0.4 discounted QALYs and increased the undiscounted life expectancy by 0.8 years per person compared to the current SOC. The average incremental cost was $6,525 per treated patient in Nigeria and $4,409 per treated patient in South Africa. The cost-effectiveness ratios were $16,302/QALY gained and $11,085/QALY gained for Nigeria and South Africa, respectively. Our results were sensitive to the probability of ART discontinuation and the unit cost for RAL.

**Conclusions:**

The combination of raltegravir and ritonavir-boosted lopinavir was projected to be cost-effective in South Africa. However, at its current price, it is unlikely to be cost-effective in Nigeria.

## Introduction

In June 2001 the United Nations issued a Declaration of Commitment to facilitate and support a global effort to combat the HIV/AIDS pandemic through a combination of prevention and treatment initiatives made universally available to all people living with HIV/AIDS (PHA). UNAIDS recently reported that since this initiative began there are encouraging signs of success, including evidence of an absolute reduction in new HIV infections [1]. UNAIDS reported that more than 6.5 million people (of a UN agreed target of 15 million by 2015) had access to combination antiretroviral therapy (ART) by the end of 2010 [1]. The majority of these individuals are receiving standard first-line ART combinations comprising of one drug selected from the non-nucleoside reverse transcriptase inhibitor (NNRTI) class with two drugs from the nucleoside/nucleotide reverse transcriptase inhibitor (N(t)RTI) class. While this approach is recommended for the initiation of ART, there is inevitable attrition. HIV ultimately develops resistance, resulting in virological failure and HIV disease progression [2]. An analysis in 2008 estimated that by 2010 between 500,000 and 800,000 people receiving first-line cART would have qualified for a switch to second-line therapy, causing the cost of second-line therapy to increase from 2% in 2006 to 35% in 2010 of the total cost [3]. Unfortunately, there is no evidence to guide how treatment of these people should be managed. The challenge of this un-met clinical need grows daily.

The current standard of care (SOC) for second-line ART consists of the introduction of a new class of ART, a ritonavir-boosted protease inhibitor, combined with two N(t)RTIs. This strategy is generally successful in settings in which virological monitoring is done 3–4 monthly, thereby minimising the selection of resistance in those considered to have virologically failed first-line ART [4]. However, in resource-limited settings most patients are managed in the absence of virological monitoring using clinical and/or immunological measures. These are neither sensitive nor specific for virological failure. As a result, when failure is detected most patients have substantial degrees of resistance to both the NNRTI and NRTI ART classes [5]–[7]. Use of agents from the N(t)RTI class in this context may contribute little to efficacy but substantially to intolerability and toxicity, particularly given their routine use in first-line ART.

Two research institutions are currently sponsoring the conduct of two independent randomised controlled trials (RCT). These both attempt to provide a firmer evidence base for guidelines for the provision of second-line ART after the failure of first-line. The Kirby Institute is conducting a non-inferiority design RCT (SECOND-LINE; NCT00931463) to compare the use of a SOC second line combination ART of ritonavir-boosted lopinavir (LPV/r) with 2−3 N(t)RTIs versus a novel nuke-sparing combination of LPV/r combined with raltegravir (RAL), the first-in-class HIV integrase strand transfer inhibitor (InSTI). The UK Medical Research Council is sponsoring the conduct of the EARNEST RCT (NCT00988039) which asks a similar question with the same agents but with a third comparator that employs LPV/r monotherapy front-end loaded with RAL for a fixed period of the first 12 weeks. Both RCTs test whether the novel combination of a boosted-PI plus RAL provides non-inferior efficacy to SOC. They also test whether it is more tolerable and less toxic using safety endpoints within the parent study as well as nested dual energy X-ray absorptiometry (DXA) sub-studies. If successful, the experimental arms (either LPV/r plus RAL or LPV/r monotherapy with front-end loaded RAL) would not require implementation of resistance testing to select second-line therapy because resistance selected in first-line would be immaterial to treatment outcomes of second-line ART.

The use of raltegravir in combination ART has been associated with less drug-related toxicity and adverse events when compared with other drug combinations currently available. It has a benign metabolic profile superior to that conferred by most other ART [8], [9]. However, regimens that include raltegravir are 6 to almost 20 times more expensive than the cost of the current first and second-line ARTs for Sub-Saharan Africa [10]. They are therefore thought to be priced out of reach for this setting. Raltegravir is currently not recommended for second-line therapy in national guidelines in Sub-Saharan Africa and is not routinely supplied or available from international groups supporting universal access to care in low and middle-income settings such as PEPFAR. Nevertheless, increased cost does not necessarily equate with diminished cost-effectiveness, particularly if the agent is associated with tangible benefits which may contribute to improved productivity and quality of life. While there are currently no published results of trials investigating LPV/r+RAL and LPV/r+2-3N(t)RTIs as a second-line therapy, we estimate the cost and likely consequences of treatment simplification with LPV/r+RAL compared to the standard of care (SOC) from published studies and collaborating sites. This methodology was inspired by a previously published simulation model projecting the long-term outcomes of treatment simplification to inform the design of a multicentre, randomised clinical trial [11]. We undertook a cost-effectiveness analysis of the application of the experimental regimen for two settings in Sub-Saharan Africa in which the RCT is itself being conducted – Nigeria (a lower-middle income country) and South Africa (an upper-middle income country).

The aim of this study was to simulate the potential risks and benefits of a novel simplification treatment strategy for second-line therapy, including cost-effectiveness, in order to help understand likely determinants of value. The results were used to calculate the incremental cost-effectiveness ratio (ICER) as the incremental cost per quality adjusted life years ($/QALY) gained from using LPV/r + RAL compared with LPV/r+2-3N(t)RTIs.

## Methodology

We developed a computer-based microsimulation model of HIV disease to evaluate the long-term outcomes for patients experiencing treatment failure of first-line ART (NNRTI +2N(t)RTIs) assigned to receive either LPV/r+RAL or LPV/r+2-3N(t)RTIs. We used decision analysis software (TreeAge Pro 2012; TreeAge Software, Boston, MA) to develop and analyse the model and perform sensitivity analyses using a 50 year time horizon from the perspective of the health-care provider. The economic model was built to simulate the likely disease progression of HIV-infected patients that were N(t)RTI and NNRTI experienced with treatment failure and unsuppressed HIV replication. Patients in the model were stratified according to CD4^+^ T-cell count, viral failure and adverse event history. Patients could transition into different health states over time based on projected long-term treatment efficacy. Weekly probabilities of clinical events including treatment failure, changes in the CD4^+^ T-cell count, adverse reactions to medications and death were used to simulate the course of disease in a hypothetical cohort of HIV-infected persons. Each state was associated with a specific treatment cost and quality of life utility. Costs and consequences were further investigated in deterministic and probabilistic sensitivity analyses. Incremental cost effectiveness ratios were expressed as 2011 US dollars per quality-adjusted life year ($/QALY) gained.

### The model

**Figure 1:**
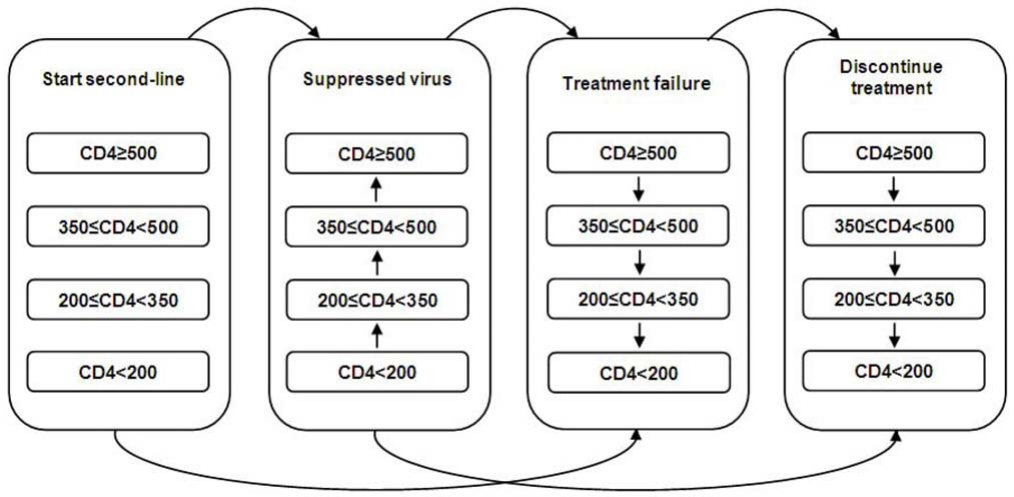
Schematic diagram of transitions through the health states in the model.

The model used in this study was based on a 16-compartment Markov model (Figure 1). Expected mean values for the base case analysis were estimated by sampling from a distribution of paths through the model's chance events in 10,000 first-order simulation trials (microsimulation). Individuals were assumed to enter the model with unsuppressed viremia and were distributed across the four possible CD4^+^ T-cell count compartments, skewed toward fewer CD4^+^ T-cells: 5%: CD4^+^≥500, 10%: 350≤CD4^+^<500, 30%: 200≤CD4^+^<350, 55%: CD4^+^<200) (Figure 1). This stratification was chosen to reflect a cohort of individuals who have failed first-line therapy by clinical and/or immunological means and who are therefore relatively immunodeficient [12].

During each weekly cycle, individuals in the model faced a series of different chance events that were dependent on the health state in which they started the cycle. Firstly, there is a probability that treatment leads to suppression of virus replication, characterised by increased CD4^+^ T-cell count. There are likewise a proportion of individuals who do not achieve suppression and move to health states characterised by losses of CD4^+^ T-cells. These people are representative of people who either have problems adhering adequately to ART or have developed drug resistance [13]. While an individual in the model remains on treatment they have a probability of experiencing a drug-related adverse event and a proportion will discontinue treatment as a consequence. A treated individual in the model also has a probability of confirmed viral rebound, at which point it is assumed that CD4^+^ T-cell count will decline. Individuals who have failed treatment virologically are assumed to remain on ART until they become immunodeficient and experience declines in CD4^+^ T-cells to below 200 cell/µL. We assume no additional treatment is supplied in the model after second-line therapy.

Treatment efficacy in our model was based on a recently published meta-analysis reporting on the rate of treatment failure among people on second-line therapy in resource-limited settings [13] (Table 1). While a SOC second-line regimen was the preferred treatment option for most studies in the analysis, we assume the treatment efficacy of LPV/r+RAL to be non-inferior to LPV/r+2-3N(t)RTIs. Therefore, the same treatment failure rates are applied to both treatment arms in the model [13]. A difference between the treatment arms was generated through the rate of drug-related adverse events and the rate of treatment discontinuation due to adverse events. For both treatment strategies, these were estimated from the BENCHMRK trials [14] (Table 1). These toxicity data were sourced for the base case analyses because the patient population is most representative of those initiating second-line therapy in resource-limited settings compared with other clinical trials and observational studies to date. However, these toxicity data are extensively analysed in sensitivity analyses (Supporting Material S3).

**Table 1:** Model transitions, and quality of life estimates.

Variable	Base case (range used in sensitivity analysis)	Distribution type	Reference
**Probability of death (HIV and non-HIV related) per year**			
CD4^+^≥350	0.007 (0.006−0.008)	Triangular	[29]
200≤CD4^+^<350	0.014 (0.011−0.017)		
CD4^+^<200	0.083 (0.033−0.406)		
**Treatment efficacy: viral suppression**			
Probability of viral suppression during first year on treatment	0.769 (0.70−0.839)	Triangular	[13]
**Probability of confirmed viral rebound during second year on treatment**	0.0467	Constant	
**Probability of confirmed viral rebound after second year on treatment**	0.155	Constant	
**Estimated probability of a drug related adverse event per year** a			
LPV/r+2-3N(t)RTIs	0.40 (0.36−0.44)	Triangular	[14]
LPV/r+RAL	0.28 (0.25−0.31)	Triangular	
**Estimated probability of discontinuing ART due to an adverse event per year** b			
LPV/r+2-3N(t)RTIs	0.044 (0.04−0.048)	Triangular	[14]
LPV/r+RAL	0.021 (0.019−0.023)	Triangular	
**CD4^+^ T-cell count increase with viral suppression while on ART (baseline CD4^+^<200) (years)**			
Increase from CD4^+^<200 to 200≤CD4^+^<350	2.80 (2.33−3.58)	Triangular	[30] ^c^
Increase from 200≤CD4^+^<350 to 350≤CD4^+^<500	3.33 (2.15−5.83)		
Increase from 350≤CD4^+^<500 to CD4^+^≥500	4.69 (3.26−8.33)		
**CD4^+^ T-cell count increase with viral suppression while on ART (baseline 200≤CD4^+^<350) (years)**			
Increase from 200≤CD4^+^<350 to 350≤CD4^+^<500	1.42 (0.9−3.38)	Triangular	[30] ^c^
Increase from 350≤CD4^+^<500 to CD4^+^≥500	3.11 (2.06−34.83)		
**CD4^+^ T-cell count increase with viral suppression while on ART (baseline 350≤CD4<500) (years)**			
Increase from 350≤CD4^+^<500 to CD4^+^≥500	2.2 (1.07−7.28)	Triangular	[30] ^c^
**CD4^+^ T-cell count reduction with viral failure while on ART (years)**			
Drop from CD4^+^≥500 to 350≤CD4^+^<500	3.2 (1.1, 5.3#)	Triangular	[31] ^d^
Drop from 350≤CD4^+^<500 to 200≤CD4^+^<350	2.1 (0.7, 3.5#)		
Drop from 200≤CD4^+^<350 to CD4^+^<200	2.2 (0.7, 3.7#)		
**CD4^+^ T-cell count reduction (not on ART) (years)**			
Drop from CD4^+^≥500 to 350≤CD4^+^<500	3.27 (3.02, 3.55)	Triangular	[32] ^e^
Drop from 350≤CD4^+^<500 to 200≤CD4^+^<350	1.96 (1.81, 2.13)		
Drop from 200≤CD4^+^<350 to CD4^+^<200	1.96 (1.81, 2.13)		
**Quality of life estimates**			
CD4^+^<200	0.702 (0.70−0.87)	Triangular	[17]–[21]
200≤CD4^+^<350	0.818 (0.78−0.94)		
350≤CD4^+^<500	0.935 (0.78−0.97)		
CD4^+^≥500	0.935 (0.88−0.97)		

a : The rates sourced from Steigbigel et al (2009) were transformed into probabilities using the Treeage RateToProb(rate; time) function. This function multiplies a rate by time, and converts it into a probability. Calculations were as follows: ratetoprob (32.8;1/100)  = 0.27963698, and ratetoprob (51.6;1/100)  = 0.403096607.

b : Calculations were as follows: ratetoprob (2.1;1/100)  = 0.020781035, and ratetoprob (4.5;1/100)  = 0.044002518.

c,d,e : Supporting Material S1.

# : Upper bound assumption.

### Costing approach

All costs are expressed as 2011 US dollars and were discounted at 3% per year for our base case scenario. We use a discount rate of 0–5% in our sensitivity analyses (Supporting Material S3). Healthcare costs were calculated using an ingredients approach where the direct medical costs of ART, opportunistic infection prophylaxis and other health resource utilisation (testing, monitoring, hospitalisation and clinic visits) are summed together to estimate the overall cost (Supporting Material S2) [15], [16]. All costs are taken from the perspective of the health-care provider.

We assume the costs of LPV/r 200/50 mg + tenofovir disoproxil fumarate and emtricitabine (TDF/FTC) 300/200 mg for the SOC in both South Africa and Nigeria. We reference Médecins Sans Frontières [10] for antiretroviral therapy costs for the Nigerian setting and the 2011 recommended retail prices for South Africa. Raltegravir is however not available in the open market in Nigeria; we estimate Raltegravir to cost $95/month. This cost represents the cost quoted by drug representatives when clinicians negotiate to have the drug brought in by the company.

### Quality-of-life

Quality of life utilities ranging between 1 (best possible health) and 0 (death) were assigned to each health state in the model and were informed by published data in the literature [17]–[21]. The health states “CD4^+^≥500” and “350≤CD4^+^<500” were mapped to utilities for asymptomatic infection, “200≤CD4^+^<350” to symptomatic infection, and “CD4^+^<200” to AIDS. Due to the large variations in estimates of utility scores, a sensitivity analysis was performed. Our base case estimates arise from a published meta-analysis of utility estimates for HIV/AIDS [17]. Quality adjusted life years were discounted at 3% per year for our base case scenario.

### Sensitivity analysis

Expected mean values were estimated by sampling from a distribution of paths through the model's chance events in 10,000 first-order simulation trials (microsimulation). We also performed probabilistic sensitivity analyses (PSA) and deterministic sensitivity analyses to examine the impact that uncertain parameters in the model had on the ICER. We conducted PSA using second-order Monte-Carlo simulations [22]. Second-order simulations capture parameter uncertainty by running a one-dimensional loop that recalculates expected values for each set of randomly sampled parameter values. After a set of simulations are run, the overall uncertainty in the model is captured by the confidence intervals around each outcome. Simulations were run over 3,000 parameter distribution values in our analysis.

## Results

### Nigeria

In the base case analysis, the reduction in treatment discontinuations estimated with LPV/r+RAL increased the undiscounted life expectancy by 0.79 years and the discounted quality-adjusted life years by 0.4 for an incremental cost of approximately $6,525 USD per person compared with the standard of care. The resulting ICER was found to be $16,302 USD per discounted quality adjusted life year gained and $10,335 USD per undiscounted life year gained (Table 2). We assume the Nigerian willingness-to-pay for an additional quality-adjusted life year is approximately $7,800; this cost-effectiveness threshold represents the 2011 GDP per capita of Nigeria (CIA World Factbook estimate in US dollars calculated using purchasing power parities) multiplied by three [23]. While assuming this to be the willingness-to-pay threshold, LPV/r+RAL would not be considered cost-effective for this setting.

**Table 2:** Incremental cost-effectiveness of LPV/r+RAL vs. standard of care.

Strategy	Discounted total cost (USD)	Discounted QALYs	Undiscounted life years	Discounted average cost-effectiveness	Discounted incremental cost-effectiveness (USD)
**Nigeria**					
Standard of care	$9,322	9.58	17.12	$973	$16,302
Raltegravir	$15,847	9.98	17.91	$1,588	
**South Africa**					
Standard of care	$19,984	9.56	17.19	$2,090	$11,085
Raltegravir	$24,393	9.96	17.98	$2,449	

**Figure 2:**
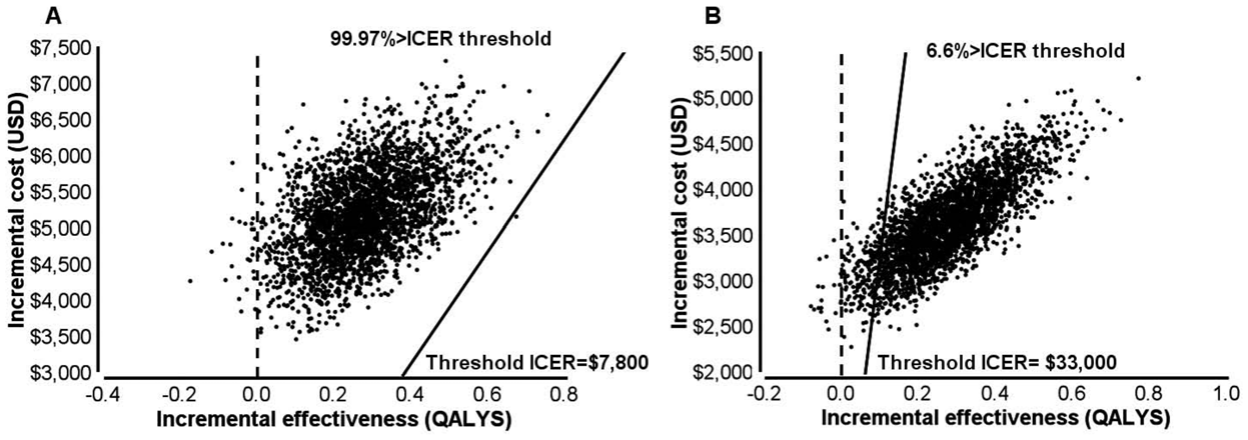
Incremental cost-effectiveness scatter plots. **A.** Nigerian setting. **B.** South African setting.

Across 3,000 PSA iterations LPV/r+RAL was more effective 99.1% of the time and more costly in all iterations. The ICER remained above the willingness-to-pay threshold in 99.97% of the iterations (Figure 2A). The mean ICER for all 3,000 iterations was found to be $18,818/QALY.

### South Africa

Access to medical care in South Africa was found to be more extensive and therefore more costly compared with Nigeria, reducing the incremental cost between SOC and LPV/r+RAL and therefore the ICER. The ICER was $11,085 USD per discounted quality adjusted life year gained and $7,554 per undiscounted life year gained (Table 2). We assume the South African willingness-to-pay for an additional quality-adjusted life year is $33,000. This estimated cost-effectiveness threshold represents the 2011 GDP per capita of the South Africa (CIA World Factbook estimate in US dollars calculated using purchasing power parities) multiplied by three. While assuming this to be the willingness-to-pay threshold, LPV/r+RAL would be considered to be cost-effective for this setting.

Across 3,000 PSA iterations conducted for the South African setting, LPV/r+RAL was more effective 99.4% of the time. LPV/r+RAL was found to be more costly in all iterations. The ICER remained lower than the willingness-to-pay threshold 93.4% of the time (Figure 2B). The mean ICER for all 3,000 iterations was $13,313/QALY.

### Deterministic sensitivity analyses

**Figure 3:**
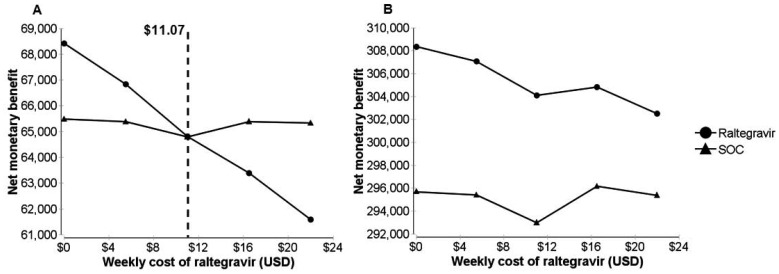
Discounted net monetary benefit. **A.** Nigerian setting. **B.** South African setting.

We conducted a series of one-way sensitivity analyses (Supporting Material S3). We found the ICER to be most sensitive to the probability of ART discontinuation and the cost of RAL. The cost of RAL in Nigeria was estimated in our base case analysis as $21.92 USD per week per person. As shown in Figure 3A, RAL is as cost-effective as SOC in Nigeria when $11.07 USD per week per person. This corresponds to an annual cost of approximately $576 USD. The price of RAL in South Africa was estimated to be $21.86 USD per week per person. As shown in Figure 3B, this price produced a higher net monetary benefit than SOC.

The WHO-preferred boosted protease inhibitors in the current guidelines are LPV/r and atazanavir/r (ATV/r). The combination of ATV/r is generally 25% cheaper than LPV/r. We therefore conducted a one-way sensitivity analysis on the cost of the boosted-PI used in the model. It was found to have no effect on the outcome of the model and only marginally reduced the overall cost (Supporting Material S3).

### Model validation

The projected outcomes produced by the model remained within the range of values reported in the literature. We compared the predicted mortality in our model with the reported mortality on second-line ART in resource-limited settings from the Ajose et al (2012) systematic review and meta-analysis [13]. Overall the unpooled discrete rates of mortality on second-line was reported to be low across all time points, 2.0–6.0% at 3 months, 5.0–10.0% at 12 months, and 5.0–7.0% at 24 months. Our model predicted a base case mortality of 3.0% at 6 months, 7.0% at 12 months, 8.0% at 24 months and 97% after 50 years.

## Discussion

This cost-effectiveness analysis predicts that a nuke-sparing combination of LPV/r+RAL is likely to be cost-effective for upper middle-income countries such as South Africa. In South Africa, the benefit associated with fewer patients discontinuing treatment, fewer patients experiencing adverse events and fewer patients experiencing HIV-disease progression events offset the increased investment in RAL. However, in Nigeria there are fewer resources for monitoring and medical care for each individual with HIV, and the resources required to provide sufficient treatment and care for PHA in Nigeria remain undersupplied. As a result there are less costly consequences for patients who are sick, making the introduction of a more costly and less toxic drug such as RAL not cost-effective.

Our analysis has a number of limitations. First and foremost, there are currently no published studies directly investigating whether there is a clinical difference between LPV/r+RAL and LPV/r+2-3N(t)RTIs among NRTI and NNRTI experienced patients. In our analysis, we assume non-inferiority. Studies to date have had mixed results. A small, single cohort study [24] conducted in HIV-infected but ART-naive participants yielded results that suggested that the combination of ritonavir-boosted darunavir and RAL may be inferior to standard first-line ART [24]. However, the PROGRESS randomised controlled trial comparing the combination of LPV/r+RAL with LPV/r+TDF/FTC suggested that the nuke-sparing drug combination offered non-inferior efficacy, safety and tolerability compared to the standard of care [25]. In addition, a DXA substudy of PROGRESS suggests a clinically relevant soft-tissue and bone toxicity advantage for the N(t)RTI-sparing strategy [26]. While no study has published clinically significant differences between drug-related adverse events, the rates of events among patients in the BENCHMRK trials are markedly different, and are what we use in our model [14].

A second limitation is the accuracy of our costing analysis. There is little data currently available reporting on the cost and utilisation of medical treatment for PHA in Sub-Saharan Africa. We therefore had unit costs and utilisation of healthcare estimated by physicians and other healthcare workers by an informal chart review in collaborating clinical sites in Nigeria. For the South African setting, unit costs were estimated from the National Health Laboratory Service and the Bio Analytical Research Corporation South Africa. Healthcare utilisation was estimated from data published in the literature [27], [28]. It is however unlikely that these estimates are far from the true cost since they represent current practice experience in the respective settings.

In conclusion, the combination of raltegravir and ritonavir-boosted lopinavir was projected to be cost-effective for an upper-middle income country in Sub-Saharan Africa. At its current price, it is unlikely to be cost-effective for lower middle-income countries such as Nigeria. However, with the additional presentation of clinical results for RAL as a second-line therapy and the entry of two new InSTIs onto the developed world market within the next 12–24 months (elvitegravir and dolutegravir) the price of RAL may be subject to competitive pressures.

## Supporting Information

Supporting Material S1 CD4 progression and viral load assumptions.
(DOC)

Supporting Material S2 Unit costs and ART utilisation assumptions.
(DOC)

Supporting Material S3 Sensitivity analyses.
(DOC)
