# Supplementary material for: The Potential Cost and Benefits of Raltegravir in Simplified Second-Line Therapy among HIV Infected Patients in Nigeria and South Africa
Source: PLoS One. 2013 Feb 15;8(2):e54435. doi: 10.1371/journal.pone.0054435 (PMC3574122; doi:10.1371/journal.pone.0054435)
Supplement: Supporting Material S1 — CD4 progression and viral load assumptions. (DOC) [file pone.0054435.s001.doc]

### Supporting material 1: CD4 progression and viral load assumptions

^c^ : Below is a summary of data from Mocroft et al. (2007) for changes in CD4^+^ count over time among people who are on effective cART.

| **CD4^+^ count at initiation of cART (cells per μL)** | **Time since starting cART (years)** | **Rate of CD4^+^ count increases per year (cells per μL) mean (95% CI)** |
| --- | --- | --- |
| ≤200 | <1 | 76 (53−99) |
|  | 1−3 | 69 (63−76) |
|  | 3−5 | 50 (36−69) |
|  | >5 | 32 (18−46) |
| 201-350 | <1 | 129 (91−166) |
|  | 1−3 | 50 (25−74) |
|  | 3−5 | 47 (24−69) |
|  | >5 | 23 (2−44) |
| >350 | <1 | 90 (37−144) |
|  | 1−3 | 50 (18−82) |
|  | 3−5 | 17 (-17−51) |
|  | >5 | 21 (-12−54) |

Abbreviations: cART, combination antiretroviral therapy; CI, confidence interval.

We use this data to estimate the average time to progress through our CD4^+^ compartments whilst on effective cART in our model. For people with undetectable viral load and who start ART with CD4^+^<200 we assume:

For CD4^+^ count increases from 0 to 200 cells per μL, average increases of 76 (53−99) cells per μL can be expected for one year then 69 (63−76) cells per μL during the first and second years. Therefore, it can be expected to take 2.80 (2.33−3.58) years to progress through this compartment.

For CD4^+^ count increases from 200 to 350 cells per μL, we have a 150 cells per µL CD4^+^ count increase. In this interval, we assume an individual has been on treatment already for approximately 2.8 years. Therefore the CD4^+^ count increases by 69 (63−76) cells per μL for 0.2 years and then by 50 (36−69) cells per μL during the third and fourth years and by 32 (18−46) cells per μL thereafter. Therefore, it can be expected to take 3.33 (2.15−5.83) years to progress through this compartment.

For CD4^+^ count increases from 350 to 500 cells per μL, we have a 150 cells per µL CD4^+^ count increase. In this interval, the CD4^+^ count increases 32 (18−46) cells per μL per year. Therefore, it can be expected to take 4.69 (3.26−8.33) years to progress through this compartment.

For people with undetectable viral load and who start ART with 200≤CD4^+^<350 we assume:

For CD4^+^ count increases from 200 to 350 cells per μL, we have a 150 cells per µL CD4^+^ count increase. Therefore the CD4^+^ count increases by 129 (91−166) cells per μL for one year and then by 50 (25−74) cells per μL for the first and second years and then by 47 (24−69) cells per μL in the third and fourth years. Therefore, it can be expected to take 1.42 (0.9−3.38) years to progress through this compartment.

For CD4^+^ count increases from 350 to 500 cells per μL, we have a 150 cells per µL CD4^+^ count increase. In this interval, we assume an individual has been on treatment already for approximately 1.42 years. Therefore the CD4^+^ count increases by 50 (25−74) cells per μL for the next 1.58 years and then by 47 (24−69) in the third and fourth years. Therefore, it can be expected to take 3.11 (2.06−34.83) years to progress through this compartment.

For people with undetectable viral load and who start ART with 350≤CD4^+^<500 we assume:

For CD4^+^ count increases from 350 to 500 cells per μL, we have a 150 cells per µL CD4^+^ count increase. Therefore the CD4^+^ count increases by 90 (37−144) cells per μL for one year. As we assume an increase to CD4^+^ count in this interval, we assume each following year is associated with an increase of 50 (18−82) cells per μL per year. Therefore, it can be expected to take 2.2 (1.07−7.28) years to progress through this compartment.

^d^ : The relationship between the CD4^+^ count slope, the patient HIV-1 RNA concentration, treatment information and demographic characteristics has been estimated by the PLATO Collaboration and led to the following regression coefficients:

| **Covariates^a^** | **Change (95% CI) in CD4^+^ count slope (cells per μL per year) - multivariate analysis** |
| --- | --- |
| Age, per 10 years older | -6.7 (-12, -1.2) |
| Female | 16.0 (-2.9, 35.0) |
| Infection via injecting drug use | -2.7 (-21,16.0) |
| Current viral load, per log_10_ copies per mL higher | -25.0 (-29.0, -20.0) |
| Current CD4^+^ count per 100 cells per μL higher | 2.2 (-2.3, 6.6) |
| Number of drugs, per additional drug | 4.8 (0.22, 9.4) |
| Boosted PI | 18.0 (7.0, 28.0) |
| Receiving ART (NNRTI) | -23.0 (-35.0, -11.0) |
| Hydroxyurea | -21.0 (-35.0, -5.8) |

^a^Constant term for multivariate model 123(82, 162) cells per μL per year

Abbreviations: ART, antiretroviral therapy; CI, confidence interval; NNRTI, non-nucleoside reverse transcriptase inhibitor; PI, protease inhibitor.

For people on treatment with detectable viral load, we assume their viral load to be ~10^3.5^ copies per mL with a median age of 35 years. Using the multivariate model, the average immunological deterioration of an individual with virological failure was determined and applied to the model. To progress through the ≥500 CD4^+^ count category we assume an average loss of 250 cells per µL CD4^+^ count in this interval; then the average CD4^+^ count slope is 123(82, 164) - 6.7(-12.0, -1.2)*3.5 + 16(-2.9, 35.0)*1 - 2.7(-21, 16.0)*0 - 25(-29.0, -20.0)*3.5 + 2.2(-2.3, 6.6)*7.5 + 4.8(0.22, 9.4)*3 + 18(7.0, 28.0)*1 - 23 (-35.0, -11.0)*0 - 21.0 (-35.0, -5.8)*0. That is, an average CD4^+^ count slope of 76.95 (-73.99, 230.50) cells per µL per year. Therefore, the average time to progress through this compartment is 3.2 (1.1, 5.3^#^) years.

To progress through the 350-500 CD4^+^ count category, we assume an average loss of 150 cells per µL CD4^+^ count in this interval; the average CD4^+^ count slope is 123(82, 164) - 6.7(-12.0, -1.2)*3.5 + 16(-2.9, 35.0)*1 - 2.7(-21, 16.0)*0 - 25(-29.0, -20.0)*3.5 + 2.2(-2.3, 6.6)*5 + 4.8(0.22, 9.4)*3 + 18(7.0, 28.0)*1 - 23 (-35.0, -11.0)*0 - 21.0 (-35.0, -5.8)*0. That is, an average CD4^+^ slope of 71.45 (-68.24, 214) cells per µL per year. Therefore, the average time to progress through this compartment is 2.1 (0.7, 3.5^#^) years.

To progress through the 200-350 CD4^+^ count category, we assume an average loss of 150 cells per µL CD4^+^ count in this interval; then the average CD4^+^ count slope is 123(82, 164) - 6.7(-12.0, -1.2)*3.5 + 16(-2.9, 35.0)*1 - 2.7(-21, 16.0)*0 - 25(-29.0, -20.0)*3.5 + 2.2(-2.3, 6.6)*3.5 + 4.8(0.22, 9.4)*3 + 18(7.0, 28.0)*1 - 23 (-35.0, -11.0)*0 - 21.0 (-35.0, -5.8)*0. That is, an average CD4^+^ slope of 68.15 (-64.79, 204.1) cells per µL per year. Therefore, the average time to progress through this compartment is 2.2 (0.73, 3.7^#^) years.

^e^: A summary of the relationship between HIV-1 RNA concentration and decline in CD4^+^ count from the prospective study by Mellors et al. (1997) is given below:

| **Plasma HIV-1 RNA concentration (copies/mL)** | **Mean decrease in CD4^+^ T cell count per year (cells/μL)** |
| --- | --- |
| ≤500 | -36.3 (-30.4, -42.3) |
| 501−3,000 | -44.8 (-39.1, -50.5) |
| 3,001−10,000 | -55.2 (-50.7, -59.8) |
| 10,001−30,000 | -64.8 (-59.6, -70.0) |
| >30 000 | -76.5 (-70.5, -82.9) |

With these data and assuming that the average viral load is ~10^4.87^ copies per mL for people without treatment, the CD4^+^ count decreases by an average of 76.5 (70.5, 82.9) per year. To progress from the CD4^+^≥500 compartment to the 350≤CD4^+^<500 compartment we assume that the average CD4^+^ count is 750 cells per μL and declines at the constant rate of 76.5 (70.5, 82.9) cells per μL each year. The average time to progress through this compartment is 250/(76.5 (70.5, 82.9)) years; that is 3.27 (3.02, 3.55) years.

To progress through the 350≤CD4^+^<500 and 200≤CD4^+^<350 compartments, we assume an average loss of 150 CD4^+^ cells per µL. The average time to progress through both compartments is 150/(76.5 (70.5, 82.9)) years; that is 1.96 (1.81, 2.13) years.

**References**

1. Mocroft A, Phillips AN, Gatell J, Ledergerber B, Fisher M, et al. (2007) Normalisation of CD4 counts in patients with HIV-1 infection and maximum virological suppression who are taking combination antiretroviral therapy: an observational cohort study. Lancet 370: 407-413.

2. Ledergerber B, Lundgren JD, Walker AS, Sabin C, Justice A, et al. (2004) Predictors of trend in CD4-positive T-cell count and mortality among HIV-1-infected individuals with virological failure to all three antiretroviral-drug classes. Lancet 364: 51-62.

3. Mellors JW, Munoz A, Giorgi JV, Margolick JB, Tassoni CJ, et al. (1997) Plasma viral load and CD4+ lymphocytes as prognostic markers of HIV-1 infection. Ann Intern Med 126: 946-954.
