# Supplementary material for: The Potential Cost and Benefits of Raltegravir in Simplified Second-Line Therapy among HIV Infected Patients in Nigeria and South Africa
Source: PLoS One. 2013 Feb 15;8(2):e54435. doi: 10.1371/journal.pone.0054435 (PMC3574122; doi:10.1371/journal.pone.0054435)
Supplement: Supporting Material S2 — Unit costs and ART utilisation assumptions. (DOC) [file pone.0054435.s002.doc]

### Supporting material 2: unit costs and health care utilisation assumptions

The unit costs and utilisation of ART are detailed below.

Unit costs for antiretroviral therapy

|  | **Units per year** | **Unit cost** | **Cost per patient per year^a^** | **Reference** |
| --- | --- | --- | --- | --- |
| **Nigeria** | | | | |
| **Standard of care** | | | | |
| Ritonavir boosted lopinavir | 1,460 | 0.31 (0.28−0.34) | 448 (402−499) |  |
| Tenofovir disoproxil fumarate and emtricitabine (truvada) | 365 | 0.37−0.86 | 174 (134−315) |  |
| Total (USD) |  |  | $622 ($536−814) |  |
| **Raltegravir based regimen** | | | | |
| Ritonavir boosted lopinavir | 1,460 | 0.31 (0.28−0.34) | 448 (402−499) |  |
| Raltegravir | 730 | 1.56 | 1140 |  |
| Total (USD) |  |  | $1588 ($1542−1639) |  |
| **South Africa** | | | | |
| **Standard of care** | | | | |
| Ritonavir boosted lopinavir | 12 | 224.88 | 2,721.05 |  |
| Tenofovir disoproxil fumarate and emtricitabine | 12 | 374.69 | 4,533.75 |  |
| Total Rand (USD) |  |  | 7,254.80 (884.73) |  |
| **Raltegravir based regimen** | | | | |
| Ritonavir boosted lopinavir | 12 | 224.88 | 2,721.05 |  |
| Raltegravir | 12 | 770.50 | 9,323.05 |  |
| Total Rand (USD) |  |  | 12,044.10 (1468.79) |  |

^a^: Unit costs for the Nigerian setting represent 2011 USD. Unit costs for the South African setting represent 2011 South African Rand. An exchange rate of 8.2 was used to adjust South African Rand to US dollars.

To account for the increased healthcare utilisation of patients with lowering immune function, healthcare costs were stratified by CD4^+^ count (CD4^+^≥500, 350≤CD4^+^<500, 200≤CD4^+^<350, and CD4^+^<200). We assume a patient with CD4^+^≥500 is asymptomatic and in good health, when CD4^+^ = 350−499 they are asymptomatic and mostly in good health, when CD4^+^ = 200−349 they have mild to moderate symptoms and when CD4^+^ <200 they have moderate to severe symptoms and are prone to disease progression to AIDS defining illnesses. Therefore, we assume patients with lower CD4^+^ counts to use more medical services and therefore accrue more cost than those with higher CD4^+^ counts.

The unit costs and utilisation of healthcare in Nigeria and South Africa estimated for this study are detailed in the tables below. For the Nigerian setting healthcare utilisation was estimated by collaborating PEPFAR sites. These estimates are derived from an unofficial chart review, clinician and other health care worker estimates and represent what a patient is charged for the services. We also had clinicians and health care workers at collaborating PEPFAR sites estimate the proportion of people receiving services for this setting.

For Nigeria, we assume CD4^+^ count to be the favoured clinical marker for disease progression rather than the more expensive HIV viral load test. In addition, while genotypic testing are available to research facilities and private practitioners, the number of patients receiving these tests are so small we assume that no patients receive these tests for both settings in our analysis. For both settings we assume the primary prophylaxis for opportunistic infections is Cotrimoxazole 960mg daily until CD4^+^ >350 for 6 months.

Undiscounted medical costs for the Nigerian setting

| **CD4**^+^ **≥500** | | | | | **Cost per patient per year, 2011 US $** | | |
| --- | --- | --- | --- | --- | --- | --- | --- |
|  |  | **Average number per year (per patient)** | **Unit cost, 2011 US $** | **% of patients receiving item per year** | **Base case** | **Low estimate** | **High estimate** |
| **Medical** | | | | | | | |
| Clinic visit | Base case | 3 | $10 | 70% | $21.00 |  |  |
|  | Low estimate | 2 | $5 | 20% |  | $2.00 |  |
|  | High estimate | 5 | $60 | 10% |  |  | $30.00 |
| **Routine laboratory** | | | | | | | |
| HIV viral load |  | 1 | $125 | 5% | $6.25 | $6.25 | $6.25 |
| Full blood examination |  | 2 | $15 | 100% | $30.00 | $30.00 | $30.00 |
| VDRL or RPR |  | 1 | $7.5 | 80% | $6.00 | $6.00 | $6.00 |
| CD4^+^ T cell lymphocyte count and percent |  | 2 | $50 | 100% | $100.00 | $100.00 | $100.00 |
| ALT |  | 1 | $13 | 100% | $13.00 | $13.00 | $13.00 |
| **Hospitalizations** | | | | | | | |
|  | Base case | 2 | $200 | 7.50% | $30.00 |  |  |
|  | Low estimate | 1 | $150 | 5% |  | $7.50 |  |
|  | High estimate | 3 | $500 | 2% |  |  | $30.00 |
| **Total cost (per year)** | Base case |  |  |  | $206.25 |  |  |
|  | Low estimate |  |  |  |  | $164.75 |  |
|  | High estimate |  |  |  |  |  | $215.25 |
| **CD4**^+^ **350-499** | | | | | | | |
| **Medical** | | | | | | | |
| Clinic visit | Base case | 3 | $10 | 50% | $15.00 |  |  |
|  | Low estimate | 2 | $5 | 40% |  | $4.00 |  |
|  | High estimate | 6 | $60 | 10% |  |  | $36.00 |
| **Routine laboratory** | | | | | | | |
| HIV viral load |  | 1 | $125 | 5% | $6.25 | $6.25 | $6.25 |
| Full blood examination |  | 2 | $15 | 100% | $30.00 | $30.00 | $30.00 |
| VDRL or RPR |  | 1 | $7.50 | 90% | $6.75 | $6.75 | $6.75 |
| CD4^+^ T cell lymphocyte count and percent |  | 3 | $50 | 100% | $150.00 | $150.00 | $150.00 |
| ALT |  | 2 | $13 | 100% | $26.00 | $26.00 | $26.00 |
| **Hospitalizations** | | | | | | | |
|  | Base case | 2 | $200 | 15% | $60.00 |  |  |
|  | Low estimate | 1 | $150 | 20% |  | $30.00 |  |
|  | High estimate | 3 | $500 | 10% |  |  | $150.00 |
| Total cost (per year) | Base case |  |  |  | $294.00 |  |  |
|  | Low estimate |  |  |  |  | $253.00 |  |
|  | High estimate |  |  |  |  |  | $405.00 |
| **CD4**^+^ **200-349** | | | | | | | |
| **Medical** | | | | | | | |
| Clinic visit | Base case | 4 | $10 | 60% | $24.00 |  |  |
|  | Low estimate | 3 | $5 | 20% |  | $3.00 |  |
|  | High estimate | 7 | $60 | 20% |  |  | $84.00 |
| **Routine laboratory** | | | | | | | |
| HIV viral load |  | 1 | $125 | 7% | $8.75 | $8.75 | $8.75 |
| Full blood examination |  | 3 | $15 | 100% | $45.00 | $45.00 | $45.00 |
| VDRL or RPR |  | 1 | $7.50 | 95% | $7.13 | $7.13 | $7.13 |
| CD4^+^ T cell lymphocyte count and percent |  | 4 | $50 | 100% | $200.00 | $200.00 | $200.00 |
| ALT |  | 2 | $13 | 100% | $26.00 | $26.00 | $26.00 |
| **Prophylaxis medication** | | | | | | | |
| Bactrim DS |  | 365 | $0.3 (0.10-0.50) | 100% | $109.50 | $36.50 | $182.50 |
| **Hospitalizations** | | | | | | | |
|  | Base case | 1 | $200 | 15% | $30.00 |  |  |
|  | Low estimate | 2 | $150 | 20% |  | $60.00 |  |
|  | High estimate | 4 | $500 | 10% |  |  | $200.00 |
| Total cost (per year) | Base case |  |  |  | $450.38 |  |  |
|  | Low estimate |  |  |  |  | $386.38 |  |
|  | High estimate |  |  |  |  |  | $753.38 |
| **CD4**^+^ **<200** | | | | | | | |
| **Medical** | | | | | | | |
| Clinic visit | Base case | 5 | $10 | 60% | $30.00 |  |  |
|  | Low estimate | 4 | $5 | 20% |  | $4.00 |  |
|  | High estimate | 10 | $60 | 20% |  |  | $120.00 |
| **Routine laboratory** | | | | | | | |
| HIV viral load |  | 1 | $125 | 7.50% | $9.38 | $9.38 | $9.38 |
| Full blood examination |  | 3 | $15 | 100% | $45.00 | $45.00 | $45.00 |
| VDRL or RPR |  | 1 | $7.50 | 90% | $6.75 | $6.75 | $6.75 |
| CD4^+^ T cell lymphocyte count and percent |  | 4 | $50 | 100% | $200.00 | $200.00 | $200.00 |
| ALT |  | 4 | $13 | 100% | $52.00 | $52.00 | $52.00 |
| **Prophylaxis medication** | | | | | | | |
| Bactrim DS |  | 365 | $0.3 ($0.10-0.50) | 100% | $109.50 | $36.50 | $182.50 |
| **Hospitalizations** | | | | | | | |
|  | Base case | 4 | $200 | 15% | $120.00 |  |  |
|  | Low estimate | 2 | $150 | 25% |  | $75.00 |  |
|  | High estimate | 6 | $500 | 5% |  |  | $150.00 |
| Total cost (per year) | Base case |  |  |  | $572.63 |  |  |
|  | Low estimate |  |  |  |  | $428.63 |  |
|  | High estimate |  |  |  |  |  | $765.63 |

### Abbreviations: ALT, alanine aminotransferase; RPR, rapid plasma reagin; VDRL, venereal disease research laboratory test

For the South African setting all diagnostic unit costs were estimates obtained from the National Health Laboratory Service and the **Bio Analytical Research Corporation South Africa. Medical and hospital costs were estimated by members of the** Desmond Tutu HIV Foundation and represent estimates of the cost to the healthcare facilities. Healthcare utilisation was estimated from data published in the literature .

Undiscounted medical costs for the South African setting

| **CD4^+^ ≥500** | | | | **Cost per patient per year, 2011 Rand $** | | |
| --- | --- | --- | --- | --- | --- | --- |
|  |  | **Average number per year (per patient)** | **Unit cost, 2011 Rand** | **Base case** | **Low estimate** | **High estimate** |
| **Medical** | | | | | | |
| Clinic visit | Base case | 10.4 | $254.00 | $2,641.60 |  |  |
|  | Low estimate | 10 | $200.00 |  | $2,000.00 |  |
|  | High estimate | 10.8 | $300.00 |  |  | $3,240.00 |
| **Routine laboratory** | | | | | | |
| HIV viral load | Base case | 1.6 | $279.87 | $447.79 | $447.79 |  |
|  | High estimate | 2 | $279.87 |  |  | $559.74 |
| Full blood examination | Base case | 1.6 | $70.43 | $112.69 | $112.69 |  |
|  | High estimate | 2 | $70.43 |  |  | $140.86 |
| VDRL or RPR |  | 0.4 | $29.93 | $11.97 | $11.97 | $11.97 |
| CD4^+^ T cell lymphocyte count and percent | Base case | 1.2 | $337.31 | $404.77 | $404.77 |  |
|  | High estimate | 1.5 | $337.31 |  |  | $505.97 |
| ALT |  | 0.8 | $37.00 | $29.60 | $29.60 | $29.60 |
| **Hospitalizations** | | | | | | |
|  | Base case | 0.4 | $2,327.45 | $930.98 |  |  |
|  | Low estimate | 0.32 | $1,745.59 |  | $558.59 |  |
|  | High estimate | 0.52 | $2,909.31 |  |  | $1,512.84 |
| Total cost (per year) | Base case |  |  | $4,579.40 ($558.46 USD) |  |  |
|  | Low estimate |  |  |  | $3,565.41 ($434.81 USD) |  |
|  | High estimate |  |  |  |  | 6,000.98 (731.83 USD) |
| **CD4^+^ 350-499** | | | | | | |
| **Medical** | | | | | | |
| Clinic visit | Base case | 10.8 | $254.00 | $2,743.20 |  |  |
|  | Low estimate | 10.6 | $200.00 |  | $2,120.00 |  |
|  | High estimate | 11.2 | $300.00 |  |  | $3,360.00 |
| **Routine laboratory** | | | | | | |
| HIV viral load | Base case | 1.6 | $279.87 | $447.79 | $447.79 |  |
|  | High estimate | 2 | $279.87 |  |  | $559.74 |
| Full blood examination | Base case | 1.6 | $70.43 | $112.69 | $112.69 |  |
|  | High estimate | 2 | $70.43 |  |  | $140.86 |
| VDRL or RPR |  | 0.4 | $29.93 | $11.97 | $11.97 | $11.97 |
| CD4^+^ T cell lymphocyte count and percent | Base case | 1.2 | $337.31 | $404.77 | $404.77 |  |
|  | High estimate | 1.5 | $337.31 |  |  | $505.97 |
| ALT |  | 0.8 | $37.00 | $29.60 | $29.60 | $29.60 |
| **Hospitalizations** | | | | | | |
|  | Base case | 0.4 | $2,327.45 | $930.98 |  |  |
|  | Low estimate | 0.32 | $1,745.59 |  | $558.59 |  |
|  | High estimate | 0.52 | $2,909.31 |  |  | $1,512.84 |
| Total cost (per year) | Base case |  |  | $4,681.00 ($570.85 USD) |  |  |
|  | Low estimate |  |  |  | $3,685.41 ($449.44 USD) |  |
|  | High estimate |  |  |  |  | $6,120.98 ($746.46 USD) |
| **CD4^+^ 200-349** | | | | | | |
| **Medical** |  |  |  |  |  |  |
| Clinic visit | Base case | 14.4 | $254.00 | $3,657.60 |  |  |
|  | Low estimate | 14 | $200.00 |  | $2,800.00 |  |
|  | High estimate | 14.8 | $300.00 |  |  | $4,440.00 |
| **Routine laboratory** | | | | | | |
| HIV viral load | Base case | 2 | $279.87 | $559.74 | $559.74 |  |
|  | High estimate | 2.5 | $279.87 |  |  | $699.68 |
| Full blood examination | Base case | 2 | $70.43 | $140.86 | $140.86 |  |
|  | High estimate | 2.5 | $70.43 |  |  | $176.08 |
| VDRL or RPR |  | 0.4 | $29.93 | $11.97 | $11.97 | $11.97 |
| CD4^+^ T cell lymphocyte count and percent | Base case | 2 | $337.31 | $674.62 | $674.62 |  |
|  | High estimate | 2.5 | $337.31 |  |  | $843.28 |
| ALT |  | 1.6 | $37.00 | $59.20 | $59.20 | $59.20 |
| **Hospitalizations** | | | | | | |
|  | Base case | 0.84 | $2,327.45 | $1,955.06 |  |  |
|  | Low estimate | 0.72 | $1,745.59 |  | $1,256.82 |  |
|  | High estimate | 0.96 | $2,909.31 |  |  | $2,792.94 |
| Non-antiretroviral medication | | | | | | |
| Co-trimoxazole 80/400mg Pack size = 500. Dose = 2 tablets/day |  | 1.5 | $95.34 | $143.01 | $143.01 | $143.01 |
| Total cost (per year) | Base case |  |  | $7,202.06 ($878.30 USD) |  |  |
|  | Low estimate |  |  |  | $5,646.23 ($688.56 USD) |  |
|  | High estimate |  |  |  |  | $9,166.14 ($1,117.82 USD) |
| **CD4^+^ <200** | | | | | | |
| **Medical** | | | | | | |
| Clinic visit | Base case | 14.8 | $254.00 | $3,759.20 |  |  |
|  | Low estimate | 14 | $200.00 |  | $2,800.00 |  |
|  | High estimate | 15.2 | $300.00 |  |  | $4,560.00 |
| **Routine laboratory** | | | | | | |
| HIV viral load | Base case | 3.6 | $279.87 | $1,007.53 | $1,007.53 |  |
|  | High estimate | 6 | $279.87 |  |  | $1,679.22 |
| Full blood examination | Base case | 4.4 | $70.43 | $309.89 | $309.89 |  |
|  | High estimate | 12.8 | $70.43 |  |  | $901.50 |
| VDRL or RPR |  | 0.4 | $29.93 | $11.97 |  |  |
| CD4^+^ T cell lymphocyte count and percent | Base case | 2.4 | $337.31 | $809.54 | $809.54 |  |
|  | High estimate | 4 | $337.31 |  |  | $1,349.24 |
| ALT |  | 2.8 | $37.00 | $103.60 | $103.60 | $103.60 |
| **Hospitalizations** | | | | | | |
|  | Base case | 3.08 | $2,327.45 | $7,168.55 |  |  |
|  | Low estimate | 2.64 | $1,745.59 |  | $4,608.36 |  |
|  | High estimate | 3.6 | $2,909.31 |  |  | $10,473.52 |
| **Non-antiretroviral medication** | | | | | | |
| Co-trimoxazole 80/400mg Pack size = 500. Dose = 2 tablets/day |  | 1.5 | $95.34 | $143.01 | $143.01 | $143.01 |
| Total cost (per year) | Base case |  |  | $13,313.30 ($1,623.57 USD) |  |  |
|  | Low estimate |  |  |  | $9,781.94 ($1,192.92 USD) |  |
|  | High estimate |  |  |  |  | $19,210.09 ($2,342.69 USD) |

### Abbreviations: ALT, alanine aminotransferase; RPR, rapid plasma reagin; VDRL, venereal disease research laboratory test

**References**

1. Médecins Sans Frontières (2011) Untangling the web of antiretroviral price reductions. 14th Edition July 2011. Geneva: Médecins Sans Frontières.

2. Cleary S, McIntyre D, Boulle A (2006) The cost-effectiveness of antiretroviral treatment in Khayelitsha, South Africa - a primary data analysis. Cost Eff Resour Alloc 4: 20.

3. Cleary S, Boulle, A., McIntyre, D., Coetzee, D. (2004) Cost-effectiveness of antiretroviral tretment for HIV-positive adults in a South African township. Cape Town: School of Public Health and Family Medicine, University of Cape Town.
