# Supplementary material for: The Potential Cost and Benefits of Raltegravir in Simplified Second-Line Therapy among HIV Infected Patients in Nigeria and South Africa
Source: PLoS One. 2013 Feb 15;8(2):e54435. doi: 10.1371/journal.pone.0054435 (PMC3574122; doi:10.1371/journal.pone.0054435)
Supplement: Supporting Material S3 — Sensitivity analyses. (DOC) [file pone.0054435.s003.doc]

### Supporting material 3: sensitivity analyses

Results of one-way sensitivity analyses.

|  | **LPV/r+RAL** | | **LPV/r+2-3N(t)RTIs** | | **Discounted incremental cost-effectiveness (USD)** |
| --- | --- | --- | --- | --- | --- |
|  | **Discounted cost (USD)** | **Discounted QALYs** | **Discounted cost (USD)** | **Discounted QALYs** |  |
| **Nigeria** |  |  |  |  |  |
| Base case | $15,847 | 9.98 | $9,322 | 9.58 | $16,302 |
| 0% Discount rate | $20,886 | 14.15 | $12,569 | 13.41 | $11,310 |
| 5% Discount rate | $13,617 | 8.24 | $8,021 | 8.00 | $23,527 |
| Cost of bPI $335 USD per year | $15,045 | 9.91 | $8,802 | 9.60 | $19,807 |
| Cost of bPI $560 USD per year | $16,262 | 9.81 | $9,795 | 9.45 | $18,237 |
| Probability of an AE on LPV/r+RAL -10% | $16,144 | 10.03 | $9,431 | 9.66 | $18,133 |
| Probability of an AE on LPV/r+RAL +10% | $15,557 | 9.84 | $9,338 | 9.56 | $22,266 |
| Probability of an AE on SOC ART -10% | $15,918 | 10.03 | $9,428 | 9.67 | $18,210 |
| Probability of an AE on SOC ART +10% | $15,578 | 9.89 | $9,227 | 9.49 | $15,613 |
| **South Africa** |  |  |  |  |  |
| Base case | $24,393 | 9.96 | $19,984 | 9.56 | $11,085 |
| 0% Discount rate | $32,804 | 13.70 | $27,450 | 13.18 | $10,217 |
| 5% Discount rate | $20,201 | 8.13 | $16,701 | 7.93 | $17,823 |
| Cost of bPI $250 USD per year | $23,639 | 9.88 | $19,385 | 9.49 | $10,963 |
| Cost of bPI $415 USD per year | $24,460 | 9.79 | $20,160 | 9.47 | $13,512 |
| Probability of an drug-related AE on LPV/r+RAL -10% | $24,211 | 9.86 | $19,725 | 9.43 | $10,463 |
| Probability of an drug-related AE on LPV/r+RAL +10% | $23,871 | 9.78 | $19,843 | 9.50 | $14,463 |
| Probability of an drug-related AE on SOC ART -10% | $24,363 | 9.95 | $20,207 | 9.70 | $16,871 |
| Probability of an drug-related AE on SOC ART +10% | $24,165 | 9.89 | $19,713 | 9.42 | $9,448 |

Abbreviations: AE, adverse event; ART, antiretroviral therapy; bPI, boosted protease inhibitor; LPV/r, ritonavir-boosted lopinavir; N(t)RTIs, nucleoside/nucleotide reverse transcriptase inhibitors; QALYs, quality adjusted life years; RAL, raltegravir; SOC, standard of care.

Further analysis on discontinuation rates:

|  | **LPV/r+RAL** | | **LPV/r+2-3N(t)RTIs** | | **Discounted incremental cost-effectiveness (USD)** |
| --- | --- | --- | --- | --- | --- |
|  | **Discounted cost (USD)** | **Discounted QALYs** | **Discounted cost (USD)** | **Discounted QALYs** |  |
| **Nigeria** |  |  |  |  |  |
| Probability of discontinuing SOC due to an AE per year | | | | | |
| 0.0 | $15,590 | 9.82 | $10,754 | 10.21 | -$12,399 (Dominated) |
| 0.014 | $15,607 | 9.88 | $10,124 | 9.95 | -$78,848  (Dominated) |
| 0.028 | $15,627 | 9.87 | $9,690 | 9.77 | $61,245 |
| 0.042 | $15,745 | 9.97 | $9,357 | 9.62 | $18,343 |
| 0.056 | $15,613 | 9.87 | $8,928 | 9.37 | $13,382 |
| 0.07 | $15,688 | 9.89 | $8,634 | 9.21 | $10,377 |
| Probability of discontinuing LPV/r+RAL due to an AE per year | | | | | |
| 0.0 | $17,359 | 10.21 | $9,278 | 9.54 | $12,060 |
| 0.014 | $16,114 | 9.96 | $9,200 | 9.47 | $14,099 |
| 0.028 | $15,326 | 9.82 | $9,308 | 9.59 | $26,399 |
| 0.042 | $14,086 | 9.46 | $9,076 | 9.36 | $50,154 |
| 0.056 | $13,649 | 9.47 | $9,238 | 9.56 | -$49,216  (Dominated) |
| 0.07 | $12,892 | 9.29 | $9,193 | 9.50 | -$18,191  (Dominated) |
| Probability of discontinuing SOC due to an AE per year | | | | | |
| 0.0 | $24,409 | 10.01 | $22,423 | 10.41 | -$4,980  (Dominated) |
| 0.014 | $24,085 | 9.86 | $21,311 | 9.98 | -$22,072  (Dominated) |
| 0.028 | $24,135 | 9.87 | $20,536 | 9.76 | $31,815 |
| 0.042 | $24,310 | 9.94 | $20,149 | 9.66 | $14,522 |
| 0.056 | $24,190 | 9.91 | $19,446 | 9.38 | $9,035 |
| 0.07 | $24,283 | 9.92 | $19,150 | 9.24 | $7,570 |
| Probability of discontinuing LPV/r+RAL due to an AE per year | | | | | |
| 0.0 | $25,797 | 10.21 | $19,990 | 9.59 | $9,378 |
| 0.014 | $24,774 | 10.03 | $19,894 | 9.55 | $10,171 |
| 0.028 | $23,553 | 9.72 | $19,680 | 9.41 | $12,479 |
| 0.042 | $23,002 | 9.66 | $19,938 | 9.56 | $29,652 |
| 0.056 | $22,274 | 9.49 | $20,001 | 9.61 | -$18,163  (Dominated) |
| 0.07 | $21,383 | 9.22 | $19,687 | 9.43 | -$8,122  (Dominated) |

Abbreviations: AE, adverse event; LPV/r, ritonavir-boosted lopinavir; N(t)RTIs, nucleoside/nucleotide reverse transcriptase inhibitors; QALYs, quality adjusted life years; RAL, raltegravir; SOC, standard of care.

**References for Supporting Material**

1. Mocroft A, Phillips AN, Gatell J, Ledergerber B, Fisher M, et al. (2007) Normalisation of CD4 counts in patients with HIV-1 infection and maximum virological suppression who are taking combination antiretroviral therapy: an observational cohort study. Lancet 370: 407-413.

2. Ledergerber B, Lundgren JD, Walker AS, Sabin C, Justice A, et al. (2004) Predictors of trend in CD4-positive T-cell count and mortality among HIV-1-infected individuals with virological failure to all three antiretroviral-drug classes. Lancet 364: 51-62.

3. Mellors JW, Munoz A, Giorgi JV, Margolick JB, Tassoni CJ, et al. (1997) Plasma viral load and CD4+ lymphocytes as prognostic markers of HIV-1 infection. Ann Intern Med 126: 946-954.

4. Médecins Sans Frontières (2011) Untangling the web of antiretroviral price reductions. 14th Edition July 2011. Geneva: Médecins Sans Frontières.

5. Cleary S, McIntyre D, Boulle A (2006) The cost-effectiveness of antiretroviral treatment in Khayelitsha, South Africa - a primary data analysis. Cost Eff Resour Alloc 4: 20.

6. Cleary S, Boulle, A., McIntyre, D., Coetzee, D. (2004) Cost-effectiveness of antiretroviral tretment for HIV-positive adults in a South African township. Cape Town: School of Public Health and Family Medicine, University of Cape Town.
